# Some Observations on the Child of To-Day

**Published:** 1914-07

**Authors:** 


**Some Observations on the Child of To-day.** In his Presidential address before the Medical Association of the Greater City of New York, New York Medical Journal, May 9, 1914, Southworth gives an outline of what has been done for the welfare of children and also of some of the things that still have to be done in this line.—C. G. L-W .

